# Long term biopsychosocial outcomes and their associations after total knee arthroplasty in individuals with knee osteoarthritis

**DOI:** 10.1038/s41598-026-47619-y

**Published:** 2026-04-09

**Authors:** Saidan Shetty, Sandeep Vijayan, Mohandas Rao KG, Sharath K. Rao, Bincy M. George

**Affiliations:** 1 Department of Anatomy, Melaka Manipal Medical College - Manipal Campus, Manipal Academy of Higher Education (MAHE), Manipal, 576104 Karnataka India; 2 Department of Orthopedics, Kasturba Medical College (KMC) Manipal, Manipal Academy of Higher Education (MAHE), Manipal, 576104 Karnataka India; 3 Division of Anatomy, Department of Basic Medical Sciences, Manipal Academy of Higher Education (MAHE), Manipal, 576104 Karnataka India

**Keywords:** Total knee arthroplasty, Knee osteoarthritis, Long-term outcomes, Biopsychosocial recovery, Psychological health, Quality of life, Follow-up, Diseases, Health care, Medical research, Rheumatology, Signs and symptoms

## Abstract

Total knee arthroplasty (TKA) is a common intervention for individuals with knee osteoarthritis (OA) aimed at reducing pain and improving function. However, recovery extends beyond surgical outcomes, involving complex interactions among physical, psychological, and social factors. This study evaluated long-term postoperative outcomes including pain, function, quality of life (QoL), and psychological status in individuals with knee OA from a biopsychosocial perspective. A cross-sectional study was conducted among patients aged 50–80 years who underwent TKA between January 2017 and December 2022 at a tertiary care hospital. Demographic and clinical information was obtained from hospital records, and patient-reported outcomes including pain, function, disease-specific and general QoL, and psychological measures were collected via telephone. Descriptive statistics and correlation analyses were performed. Of 810 eligible patients, 492 responded. While 80.4% reported satisfaction postsurgery, only 28% attended long-term follow-up. The mean pain score was 4.79 ± 3.2, and the lower extremity functional scale score was 43.5 ± 5.8. The QoL scores reflected overall improvement. However, 25.8% reported kinesiophobia, 17.3% reported pain catastrophizing, 17.6% reported elevated scores on the Central Sensitization Inventory, 5.6% reported depressive symptoms, 9.1% reported high stress, and 6.1% reported low self-efficacy. Significant correlations were observed between pain, function, and QoL scores and psychological variables after TKA. Despite overall favorable outcomes, some patients experienced persistent pain, functional limitations, and psychological distress. Comprehensive, biopsychosocial-informed follow-up is essential to optimize long-term recovery after TKA. The study protocol was registered with the Clinical Trial Registry-India (CTRI/2022/12/048118) on 14 December 2022.

## Background

Osteoarthritis is one of the most prevalent musculoskeletal conditions worldwide and is a major contributor to chronic pain, limited mobility, and long-term disability. In India, osteoarthritis is the most common joint disorder, with reported prevalence rates ranging from 22 to 39%^1–3^. The disease is characterized by gradual degeneration of the articular cartilage and associated structural changes in the subchondral bone and periarticular tissues. These pathological alterations result in substantial functional limitations, often affecting individuals’ ability to perform daily tasks^4–8^.

Total knee arthroplasty (TKA) is frequently recommended for patients with end-stage knee osteoarthritis who do not respond to conservative treatment^9,10^. While early postoperative outcomes and quality of life are largely shaped by pain and disability^5,11^, patients often report notable improvements in function and quality of life in the months and years following surgery, largely due to sustained pain relief and enhanced mobility^12–14^. Despite these overall benefits, various sociodemographic, physical, and psychological factors can continue to impact quality of life outcomes after TKA^15,16^.

To fully understand the impact of TKA, it is important to move beyond a narrow biomedical view and adopt a broader biopsychosocial perspective that incorporates physical, psychological, and social dimensions of recovery^17,18^. Although TKA is widely recognized for its effectiveness in relieving pain and restoring joint function, patient outcomes in the long- term can be highly variable^19^. These variations are shaped not only by surgical or clinical factors but also by ongoing impairments, emotional responses, and the individual’s ability to reintegrate into daily life. Many patients continue to experience discomfort, functional challenges, or dissatisfaction well beyond the immediate postoperative phase, highlighting the need for comprehensive evaluation over extended periods^19,20^.

Psychological and social influences play critical roles in determining long-term recovery trajectories following TKA^21,22^. Emotional factors such as fear of movement, negative pain beliefs, low self-efficacy, and depressive symptoms can persist for years after surgery, diminishing the overall benefits of pain relief and joint replacement^22–26^. Additionally, social determinants including occupational demands, family support, and access to rehabilitation services, can significantly affect participation in everyday and community activities^27–30^. These aspects of recovery often remain underexplored, even though they may explain why some individuals fail to achieve optimal functional independence despite technically successful surgery^18^.

There remains a considerable gap in the literature regarding long-term, multidimensional recovery after TKA, particularly within low- and middle-income countries such as India^2,31^. Sociocultural expectations, physically demanding lifestyles, and disparities in healthcare accessibility may influence outcomes in ways that differ from those in high-income settings. However, few studies have addressed these factors comprehensively or through a biopsychosocial lens^28,30,32,33^. This study seeks to address that gap by evaluating long-term postoperative recovery in individuals with knee osteoarthritis who have undergone TKA. By considering pain, function, psychological health, and social reintegration collectively, this investigation aims to contribute meaningful insights into the complex nature of sustained recovery and patient well-being following TKA.

The primary aim of this study was to evaluate pain, function, quality of life, and psychological outcomes following TKA in individuals with knee OA. A secondary objective was to examine the associations between psychosocial variables (kinesiophobia, pain catastrophizing, central sensitization, depression, perceived stress, and self-efficacy) and pain, functional status, and quality of life outcomes in the long-term postoperative period.

## Methods

### Study design and participants

This is a cross-sectional study performed at the Department of Orthopedics of a tertiary care hospital in South India. Ethical approval was obtained from the Institutional Ethics Committee (IEC) (IEC1-180-2022), and the study protocol was registered with the Clinical Trial Registry-India (CTRI/2022/12/048118) (http://ctri.nic.in/) on 14 December 2022. The study was conducted in accordance with the Code of Ethics of the World Medical Association (Declaration of Helsinki). The reporting of this study adhered to the STROBE Statement (Strengthening the Reporting of Observational Studies in Epidemiology) guidelines for cross-sectional studies.

The inclusion criteria were participants aged 50 to 80 years of either sex who were diagnosed with primary knee osteoarthritis and who underwent TKA from January 2017 to December 2022 at a tertiary care hospital. The exclusion criteria were as follows: (1) mortality; (2) unreachability through telephone; (3) unwillingness to participate in the study; and (4) inflammatory arthritis affecting multiple joints, neurological deficits, or posttraumatic arthritis.

### Instrumentation and procedure

The hospital records of 810 patients admitted for TKA were obtained from the hospital medical records directory, and their contact numbers were obtained. Patient screening was performed based on the inclusion and exclusion criteria. The demographic, socioeconomic, and medical/surgical details were collected from hospital records and through telephonic surveys. The patients were contacted over the telephone, a trained therapist explained the study in their preferred language (English/Hindi/Kannada/Tulu), and telephonic consent was obtained from the patients. After providing consent, the time for the interview was fixed. A telephonic survey of the patients was performed using the validated data collection form and standard questionnaires. The postoperative rehabilitation history, present complaints, and current physiotherapy treatment (if any) were obtained from the patient him/herself.

### Demographic, socioeconomic, and medical/surgical details

The details collected included age (in years), sex (men/women), body mass index (in kg/m²) at the time of surgery, unilateral or bilateral, distance between hospital and residence, occupation, availability of health insurance, surgical details, past medical history of comorbidities, and duration since surgery. The patient’s postoperative rehabilitation history, present complaint, and current exercise regimen were obtained.

### Pain, function, and quality of life assessment

Pain severity was evaluated using the Numerical Pain Rating Scale (NPRS), which ranges from 0 to 10^34,35^. Functional activities were assessed using a self-structured questionnaire that included information about the ability to perform functional activities and social participation. The Lower Extremity Functional Scale (LEFS) was utilized to assess patients’ initial function, monitor ongoing progress, and evaluate outcomes for those with lower extremity issues. The LEFS has excellent internal reliability, with a Cronbach’s alpha of 0.96^36^.

The Knee Injury and Osteoarthritis Outcome Score (KOOS) was used to evaluate disease-specific quality of life^37,38^. It includes five distinct subscales - pain, other symptoms, activities of daily living, function in sport and recreation, and knee-related quality of life, each scored separately. The test-retest reliability for all the subscales is substantial to excellent, with intraclass correlation coefficients (ICCs) ranging from 0.78 to 0.91^37,38^. The Short Form 36 Health Survey Questionnaire (SF-36) questionnaire was used to evaluate overall quality of life^39–41^. It comprises eight scales: physical functioning, role physical, bodily pain, general health, vitality, social functioning, role emotional, and mental health. The minimum clinically important difference (MCID) for the physical domains of the SF-36 ranges from 11.56 for physical function to 16.86 for bodily pain^39–41^.

### Psychological outcome assessment after TKA in individuals with knee osteoarthritis

Fear of movement was assessed using the Tampa Scale of Kinesiophobia (TSK), a self-report questionnaire that evaluates fear of movement, fear-avoidance behaviors, and fear related to physical activity^42,43^. Pain catastrophizing was evaluated using the Pain Catastrophizing Scale (PCS), a self-administered questionnaire that measures exaggerated negative thoughts and feelings related to actual or anticipated pain^44,45^. The Central Sensitization Inventory (CSI) was used to assess central sensitization, a physiological phenomenon in which dysfunction in the central nervous system leads to neuronal dysregulation and increased excitability, causing heightened sensitivity to both painful and nonpainful stimuli^46^. Central sensitization is considered a risk factor for ongoing postoperative pain and patient dissatisfaction following TKA^47^. The CSI has strong reliability and validity, with a test-retest reliability of 0.82 and a Cronbach’s alpha of 0.88^46,48^.

Depression was evaluated using the Patient Health Questionnaire (PHQ), specifically the PHQ-9 depression module, which rates each of the nine Diagnostic and Statistical Manual of Mental Disorders (DSM)-IV criteria on a scale from “0” (not at all) to “3” (nearly every day). The PHQ has strong reliability, with Cronbach’s alpha values ranging from 0.81 to 0.90. It has shown a sensitivity of 88% and specificity of 88% for identifying major depression^49^. Stress was evaluated using the Perceived Stress Scale (PSS), a 10-item questionnaire designed to assess the level of stress experienced by individuals based on their feelings and thoughts over the past months. Each item is scored on a scale from 0 (never) to 5 (very often), with the total score ranging from 0 to 40. The internal consistency reliability of the PSS-10 total score and subscale scores was good. The internal consistency analysis revealed strong agreement for both the PSS Factor-1 and PSS Factor-2 scores, with Cronbach’s alpha values of 0.78 and 0.71, respectively, indicating an acceptable level of consistency^50,53^.

Self-efficacy was measured using the Arthritis Self-Efficacy Scale (ASES), a widely utilized self-report tool that evaluates an individual’s confidence in managing pain and functioning despite arthritis-related symptoms. The scale demonstrates strong internal consistency, with alpha coefficients ranging from 0.75 to 0.95 (mean α = 0.88). Test-retest reliability (r) showed stability coefficients ranging from *r* = 0.56 over a six-month interval to *r* = 0.90 over three months, with an average of *r* = 0.86^52^. Internal consistency was strong across multiple metrics, with Cronbach’s alpha ranging from 0.87 to 0.94, omega coefficients from 0.87 to 0.93, and the greatest lower bound estimates between 0.90 and 0.95^53^. Validated versions of all questionnaires were used in the respective languages of administration to ensure cultural and linguistic appropriateness and to minimize measurement bias.

### Participant review

In accordance with the responses and problems faced by the participants, appropriate individualized home programmes were advised based on the impairments, activity limitations, and participation restrictions. It included home exercises, do’s and don’ts following TKA, preventive strategies, advice to prevent the development of osteoarthritis on the other extremity, strategies to improve the performance of activities of daily living and participation in society and psychological support wherever needed. Individuals who required additional support were taken care of via virtual meetings whenever needed. Regular group virtual meetings were organized to help individuals ask their queries if any, provide advice regarding exercise and physical activity, and monitor them.

### Data analyses

Data were analyzed using Jamovi version 2.6.23. Descriptive statistics, including means, standard deviations, frequencies, and percentages, were used to summarize demographic characteristics, pain (NPRS), function (LEFS), quality of life (KOOS and SF-36), and psychological outcomes (TSK, PCS, CSI, PHQ-9, PSS, and ASES). Associations between pain, function, and quality of life measures with psychological outcomes were assessed using Spearman’s rank correlation coefficients. To control for Type I error due to multiple testing, Bonferroni correction was applied within each clinical outcome domain. The adjusted significance threshold was set at *p* < 0.008. Correlations meeting this threshold were considered statistically significant. To control for potential confounding factors, multivariate linear regression analyses were performed for each primary outcome (pain, function, and quality of life) with psychological variables entered as independent predictors.

To examine the influence of socioeconomic and environmental factors on long-term postoperative outcomes, Mann-Whitney U tests were applied for binary variables, while Spearman correlations were used for ordinal and continuous variables. Clinical outcomes included pain (NPRS), function (LEFS), and quality of life (KOOS and SF-36), and psychological outcomes included TSK, PCS, CSI, PHQ-9, PSS, and ASES. Bonferroni-adjusted thresholds applied where appropriate.

## Results

### Demographics

A total of 810 patients who underwent TKA from January 2017 to December 2022 at the tertiary care hospital were contacted via telephone. The standard medial parapatellar retinacular approach was employed for TKA for all participants. Four hundred ninety-two individuals received the call, and 318 individuals could not be contacted either because of invalid numbers, calls were not answered, telephone numbers were not in service, or numbers were not reachable. The mean age of the participants was 65.2 ± 8.2 years. Among the 492 participants, 73.4% were women, and 26.6% were men. The mean body mass index was 27.6 ± 8.8 kg/m^2^ at the time of surgery (Table 1).

**Table 1:** Demographic and anthropometric characteristics of the participants.

Variables	Mean ± SD (*n* = 492)
Age (in years)	65.2 ± 8.2
Gender (women: men)	361:131
Side operated (Bilateral: Unilateral)	113:379
Body mass index (in kg/m^2^) at the time of surgery	27.6 ± 8.8
Duration of knee pain before surgery (in months)	22.3 ± 8.9

SD: standard deviation; kg/m^2^: kilogram per meter square.

The average duration of knee pain before surgery was 22.3 ± 8.9 months. A total of 64% of the individuals reported having comorbidities such as hypertension and diabetes mellitus. A total of 22.7% of individuals underwent bilateral TKA, and 77.2% underwent unilateral TKA. All participants received multiple sessions of inpatient physiotherapy during their hospital stay following TKA. With regard to postoperative safety outcomes, no major postoperative complications such as surgical site infections were reported among the respondents at the time of assessment. Nineteen participants (3.86%) reported having undergone revision surgery within the follow-up timeframe after the index TKA procedure performed a few years earlier.

### Social factors

The percentage of individuals who had health insurance was 18%. A total of 74.7% of the individuals were at home and not involved in any occupation. The remaining 25.4% (*n* = 125) of the individuals reported that they practice agriculture, business, shopkeeping, and manual labor. A total of 70.9% (*n* = 349) lived more than 30 km away from the hospital. 65% of individuals (*n* = 320) mentioned that they perform some form of physical activity, especially walking. A total of 80.4% of the patients (*n* = 396) were satisfied after surgery, and 19.6% (*n* = 96) were unsatisfied after surgery.

A total of 28% of the individuals (*n* = 138) came to the hospital for long-term follow-up after 6 months following TKA, and 72% of the individuals (*n* = 354) were lost to follow-up. Among the 138 individuals who underwent long-term follow-up, 30.4% came to the hospital for consultation for problems other than knee OA. The patient-reported reasons for not completing follow-up after TKA varied. The most reported reason was the absence of pain or other symptoms, as 132 individuals (37.2%) did not feel the need to return for follow-up. A total of 56 individuals (15.8%) reported long travel distances to the hospital as a barrier, whereas 32 patients (9%) reported that perceived long waiting times discouraged them from attending. Thirty-one individuals (10.4%) missed follow-up due to the impact of the COVID-19 pandemic, and 29 (8.1%) did not have a caregiver to accompany them. Additionally, 27 patients (7.6%) experienced economic difficulties that prevented them from affording charges related to the visit. A small proportion (7%) did not specify any reason. These findings reflect a combination of personal, logistical, and systemic challenges influencing follow-up adherence in patients post-TKA.

Among individuals who were lost to follow-up (*n* = 354), 56.2% (*n* = 199) stayed at a distance > 30 km from the hospital, 14.9% (*n* = 53) were not satisfied with the surgery and its outcomes, 30.8% (*n* = 109) had moderate to severe pain (NPRS > 6), 18.9% (*n* = 67) had moderate to severe functional limitations (LEFS scores in the range 20–40), 18% (*n* = 64) had extreme knee problems according to the KOOS (score < 25), and 20.9% (*n* = 74) had poor SF-36 scores (score < 25).

### Pain, function, and quality of life assessment after TKA in individuals with knee OA

The mean postoperative pain score on the NPRS was 4.79 ± 3.2. Approximately 28% (*n* = 138) of the individuals reported experiencing chronic pain in the long- term following TKA. The pain was predominantly described as dull and aching in nature, with intermittent episodes of sharp or shooting sensations, particularly during movement or weight-bearing activities. Many participants reported a fluctuation in pain intensity over a 24-hour period, with increased discomfort typically occurring in the evenings or after prolonged activity. Some patients also experienced stiffness and discomfort at rest.

The mean LEFS score was 43.5 ± 5.8. The majority of individuals (*n* = 413, 83.9%) reported improved functional status in the long-term following TKA, with many (*n* = 345, 70.12%) experiencing better mobility and greater ease in performing daily activities such as walking, standing, and using stairs. Despite these overall improvements, a subset of patients (*n* = 79, 16.05%) continued to experience functional limitations, particularly during more demanding activities or prolonged physical exertion. Some (*n* = 49, 9.95%) reported the use of compensatory movement patterns or reliance on assistive devices to manage residual difficulties. Among those who were involved in an occupation (*n* = 125, 25.4%), 64.8% (*n* = 81) reported returning to roles in agriculture, business, shopkeeping, and manual labor. While they were able to resume work, many (*n* = 56, 44.8%) experienced aggravated pain during physically demanding tasks. Most individuals (*n* = 381, 77.4%) reported satisfactory participation in family and community activities following TKA, with an improved ability to engage in social interactions and gatherings. However, few (*n* = 65, 13.2%) individuals expressed limitations in attending events that required prolonged standing and stair climbing, indicating that residual physical challenges continued to affect full social reintegration.

The mean KOOS score was 79.2 ± 14.8 post-TKA (pain: 85.1 ± 16.1, symptoms: 81 ± 14.9, activities of daily living: 86.3 ± 15.2, quality of life: 70.5 ± 22.7), and the mean SF-36 score was 67 ± 20.1 post-TKA (physical performance: 66.2 ± 22.1, physical role: 74.9 ± 23.1, mental health: 70.8 ± 26.8, vitality: 54.2 ± 19.2, emotional role: 64.5 ± 24.2, social role: 58.2 ± 21.9, physical pain: 56.4 ± 32.8, general health: 78 ± 20.1, mental component score: 52.3 ± 9.8, physical component score: 45.3 ± 8.6).

### Psychological status after TKA in individuals with knee OA

Among the 492 individuals assessed, 25.8% (*n* = 127) presented high levels of kinesiophobia as measured by the TSK score of ≥ 37. Additionally, 17.3% (*n* = 85) scored above the clinical threshold on the PCS (≥ 30), whereas 17.6% (*n* = 87) demonstrated signs of central sensitization according to the CSI (≥ 40). Depressive symptoms, identified using the PHQ (≥ 10), were present in 5.6% (*n* = 28) of the cohort. Furthermore, 9.1% (*n* = 45) reported high levels of perceived stress on the PSS, and 6.1% (*n* = 30) reported low arthritis self-efficacy based on the ASES. These findings underscore the presence of various psychological and pain-related factors within the population.

### Correlations of pain, function, and quality of life with psychological outcomes after TKA in individuals with knee OA

A correlational analysis revealed statistically significant positive associations between NPRS scores and psychological outcomes, including kinesiophobia (ρ = 0.42), pain catastrophizing (ρ = 0.45), and central sensitization (ρ = 0.48). Moderate correlations were also noted with depressive symptoms (PHQ-9: ρ = 0.32) and perceived stress (PSS: ρ = 0.30). Arthritis self-efficacy (ASES) was statistically significantly negatively correlated with pain scores (ρ = −0.38), indicating that patients reporting greater pain had lower self-efficacy (Table 2). These results underscore the complex interplay between the physical and psychological components of pain in the long-term recovery phase post-TKA.

**Table 2:** Spearman’s rank correlation coefficients (ρ) between psychological outcomes and clinical variables, including pain (NPRS), physical function (LEFS), and quality of life (KOOS and SF-36 subscales).

Psychological outcomes	Pain (NPRS)	Function (LEFS)	Quality of life (KOOS - Total)	Quality of life (SF-36 - PCS)	Quality of life (SF-36 - MCS)
Kinesiophobia (TSK)	0.521	– 0.467	– 0.543	– 0.498	– 0.421
Pain catastrophizing (PCS)	0.498	– 0.441	– 0.507	– 0.469	– 0.398
Central Sensitization (CSI)	0.476	– 0.428	– 0.496	– 0.439	– 0.386
Depression (PHQ-9)	0.409	– 0.393	– 0.454	– 0.411	– 0.524
Perceived stress (PSS)	0.432	– 0.401	– 0.471	– 0.426	– 0.549
Self-efficacy (ASES)	-0.387	0.512	0.524	0.487	0.463

NPRS = Numeric Pain Rating Scale; LEFS = Lower Extremity Functional Scale; KOOS = Knee Injury and Osteoarthritis Outcome Score (Total score); SF-36 PCS = Short Form-36 Physical Component Summary; SF-36 MCS = Short Form-36 Mental Component Summary; TSK = Tampa Scale for Kinesiophobia; PCS = Pain Catastrophizing Scale; CSI = Central Sensitization Inventory; PHQ-9 = Patient Health Questionnaire-9; PSS = Perceived Stress Scale; ASES = Arthritis Self-Efficacy Scale. Spearman’s rank correlation coefficients (ρ) are presented. Given multiple comparisons, Bonferroni correction was applied and correlations with *p* < 0.008 were considered statistically significant. All reported correlations met this adjusted threshold.

A moderate negative correlation was found between LEFS and TSK (ρ = − 0.46, *p* < 0.001), PCS (ρ = − 0.38), and CSI (ρ = − 0.40), suggesting that individuals with greater psychological distress had lower functional status. The LEFS also showed mild negative correlations with the PHQ-9 (ρ = − 0.31) and PSS (ρ = − 0.28), indicating that higher levels of depressive symptoms and perceived stress were associated with reduced function. Conversely, LEFS had a moderate positive correlation with ASES (ρ = 0.44), implying that greater self-efficacy was associated with better functional outcomes (Table 2).

All the KOOS subdomain scores were statistically significantly negatively correlated with psychological distress. The KOOS pain subscale was negatively correlated with the TSK (ρ = − 0.43), PCS (ρ = − 0.41), and CSI (ρ = − 0.39) scores. The KOOS activities of daily living and quality of life subscales demonstrated moderate negative correlations with the PHQ-9 (ρ = − 0.33 and − 0.35, respectively) and PSS (ρ = − 0.30 and − 0.32). The KOOS quality of life subscale showed the strongest positive correlation with the ASES score (ρ = 0.42), suggesting that patients with greater confidence in managing their arthritis reported better knee-related quality of life (Table 2).

The SF-36 physical and mental health domains also showed consistent associations with psychological variables. The physical component summary (PCS) and role physical domains were moderately and negatively correlated with the TSK (ρ = − 0.40), PCS (ρ = − 0.38), and CSI (ρ = − 0.37) scores. The mental component summary (MCS) score was statistically significantly negatively correlated with the PHQ-9 score (ρ = − 0.49) and the PSS score (ρ = − 0.45), indicating worse mental health with greater depressive symptoms and perceived stress. Positive correlations were observed between both the PCS and MCS scores of the SF-36 and the ASES (ρ = 0.40 and 0.48, respectively), indicating that greater self-efficacy was associated with improved physical and mental quality of life (Table 2).

### Associations of socioeconomic, environmental, and occupational factors with long-term outcomes after TKA in individuals with knee OA

Socioeconomic and environmental factors, including health insurance status, urban versus rural residence, education level, and distance from the hospital, were evaluated for potential associations with long-term clinical and psychological outcomes. No statistically significant relationships were observed between health insurance status, urban/rural residence, or education level and pain (NPRS), function (LEFS), quality of life (KOOS, SF-36), or psychological outcomes. Distance from the hospital showed a weak negative correlation with SF-36 physical and mental component scores (ρ = − 0.12 to − 0.15), suggesting that greater travel distances may slightly impact perceived quality of life, although the effect size was small. Occupational background showed a weak to moderate association with functional outcomes measured by the LEFS. Individuals in physically demanding roles tended to report slightly lower functional scores than those in less demanding occupations, suggesting that the physical demands of work may modestly influence long-term functional recovery following TKA. Overall, these findings indicate that long-term outcomes after TKA were largely independent of socioeconomic and environmental factors in this cohort.

## Discussion

This study aimed to evaluate pain, function, quality of life, and psychological outcomes following TKA in individuals with knee OA.

### Pain assessment after TKA in individuals with knee OA

The majority of patients experienced improvements in pain levels, functional outcomes, and health-related quality of life following TKA^11,54,55^. The mean postoperative pain score on the NPRS was 4.79 ± 3.2, indicating that while the majority of patients experienced substantial improvements in pain levels, a subset (28%) continue to face challenges related to long-term persistent postsurgical pain for 12 months or longer following TKA^18,58,57^. Hence, it is important to evaluate long-term pain following TKA.

Pain often follows a fluctuating 24-h pattern, worsening in the evenings or after prolonged activity, and occasionally occurs at rest, suggesting the possible involvement of central sensitization or neuropathic components. Importantly, pain perception in these individuals may be influenced by multiple factors, such as comorbid conditions, preoperative pain levels, psychological distress, and socioenvironmental influences^58,59^. In the present cohort, the mean duration of knee pain prior to surgery indicated prolonged nociceptive exposure. Such chronic preoperative symptoms may contribute to central sensitization and could partly explain the persistence of pain in a subset of individuals following TKA.

Factors such as prosthesis-related complications, compensatory movement strategies, and low-grade inflammation may also contribute to persistent discomfort. Furthermore, psychological elements such as pain catastrophizing, depression, and kinesiophobia can amplify the perception of pain and limit functional recovery^24,44,60^. These findings highlight the persistence of pain in a subgroup of patients even after surgical intervention, emphasizing the importance of long-term monitoring to identify at-risk individuals and address unresolved issues that may hinder full recovery^18,61,62^.

### Functional status after TKA in individuals with knee OA

The findings of this study suggest that most of the individuals demonstrated improved functional status in the long- term following TKA, as reflected by a mean LEFS score of 43.5 ± 5.8^65^. This score falls within the range suggestive of mild to moderate functional limitations, which supports the literature that most patients benefit from statistically significant pain relief and improved mobility post-TKA. Increased ease in performing daily activities such as walking, standing, and stair climbing likely contributes to a greater sense of independence and quality of life. However, persistent limitations reported by a subset of patients, especially during high-demand activities, highlight that functional recovery may not be uniform. Factors such as muscle weakness, altered biomechanics, residual joint stiffness, or pain exacerbation during exertion may hinder full restoration of function in some individuals^64,65^. Additionally, the use of compensatory strategies or assistive devices among some patients indicates ongoing physical adaptations to address incomplete biomechanical recovery^68,67^.

Occupational reintegration was noted among patients involved in agriculture, shopkeeping, and manual labor, but physically demanding tasks often triggered pain, potentially limiting work endurance and productivity. This might also be due to arthritic involvement of the other limb^68,69^. This underscores the importance of tailoring rehabilitation to the occupational demands of patients. Social participation was largely positive, with most individuals reporting improved engagement in family and community life. Nonetheless, a few participants still experienced difficulties attending events requiring prolonged standing or stair use, suggesting that physical limitations may persist in socially demanding contexts. These observations reinforce the need for function-oriented, long-term rehabilitation programs that address not only pain and mobility but also the real-world challenges of returning to work and social environments^29,70^. A more holistic recovery strategy encompassing functional, occupational, and social goals may be essential for achieving optimal outcomes post-TKA.

### Quality of life after TKA in individuals with knee OA

After TKA, individuals with knee osteoarthritis often experience statistically significant medium- to long-term improvements in their quality of life, largely attributed to reduced pain and enhanced functional ability^12,15^. The duration of quality of life follow-up assessments following TKA ranges from short-term to long-term to adequately track the trajectory of recovery^14^. The mean KOOS score was 79.2 ± 14.8 post-TKA. KOOS scores are converted to a scale of 0–100, where 0 indicates severe knee issues and 100 signifies no knee problems at all^37,73^. Our study revealed higher KOOS scores following TKA. The mean SF-36 score was 67 ± 20.1 post-TKA. Higher SF-36 scores reflect better health status, with a mean score of 50 established as a normative benchmark across all scales^72,73^.

### Psychological status after TKA in individuals with knee OA

The present study highlights the notable prevalence of psychological and pain-related factors among individuals following TKA. Most prominently, more than one- quarter (25.8%) of the cohort exhibited high levels of kinesiophobia, reflecting a persistent fear of movement and reinjury that can adversely affect recovery. This finding is clinically significant, as heightened kinesiophobia has been associated with delayed rehabilitation outcomes, reduced physical activity, and lower functional recovery. The persistence of such fear despite surgical intervention may indicate the need for integrated psychosocial support alongside physical rehabilitation to optimize postoperative outcomes^24,74–76^.

These findings also indicate that many patients exhibit maladaptive pain processing and psychological distress. Specifically, 17.3% reported elevated pain catastrophizing, and 17.6% reported signs of central sensitization. Pain catastrophizing, characterized by magnification and helplessness, is known to amplify pain perception and may hinder recovery^77–79^. Similarly, central sensitization, characterized by a hypersensitive nervous system, has been implicated in persistent postoperative pain and poor functional improvement. These observations reinforce the growing understanding that chronic pain mechanisms are not solely nociceptive but are influenced by complex biopsychosocial interactions^80–82^.

Although depressive symptoms (5.6%) and high stress (9.1%) were less prevalent, even mild distress may impair engagement in rehabilitation and coping ability and worsen pain perception. Importantly, stress and depression are modifiable risk factors, and early identification through tools such as the PHQ and PSS may allow for timely psychosocial interventions that improve overall recovery trajectories^60,83–85^.

The finding that 6.1% of the patients had low arthritis self-efficacy highlights the importance of patients’ belief in their ability to manage symptoms and engage in self-care activities. Low self-efficacy has been linked to poorer functional outcomes, higher levels of pain, and reduced adherence to rehabilitation protocols. These results suggest that self-management strategies, patient education, and confidence-building interventions should be integral parts of postoperative care^86–89^.

Prolonged preoperative pain may also negatively influence psychological factors, including reduced self-efficacy, maladaptive coping strategies, and fear-avoidance behaviors. Therefore, pain chronicity prior to surgery may have contributed to the biopsychosocial profiles observed in this study. Overall, this study emphasizes the need for a multidimensional approach to TKA rehabilitation that targets not only physical recovery but also psychological resilience and cognitive coping strategies.

### Correlations of pain, function, and quality of life with psychological outcomes after TKA in individuals with knee OA

The findings of this study provide compelling evidence that psychological factors remain statistically significantly related to physical outcomes following TKA, especially in the long-term recovery phase. Through the exploration of correlations between pain, function, and quality of life with psychological variables, our results support the biopsychosocial framework, which suggests that recovery after orthopedic surgery is influenced by a complex interplay of physical, emotional, and cognitive factors. To strengthen the validity of our findings, all correlation results were adjusted for multiple comparisons using the Bonferroni correction, and multivariate analyses confirmed that psychological variables independently contributed to long-term pain, functional, and quality of life outcomes, even after controlling for demographic, surgical, and occupational factors.

### Pain and psychological outcomes after TKA in individuals with knee OA

Pain intensity, as measured by the NPRS, was statistically significantly correlated with several key psychological constructs. Higher pain levels were positively associated with increased kinesiophobia (TSK), pain catastrophizing (PCS), central sensitization (CSI), depressive symptoms (PHQ-9), and perceived stress (PSS) but inversely related to arthritis self-efficacy (ASES). These findings highlight the reinforcing cycle between persistent pain and maladaptive psychological patterns. Patients with heightened fear of movement or exaggerated pain-related thoughts may develop avoidance behaviors and hypervigilance, creating a feedback loop that intensifies pain and delays recovery^24,78,81^.

Notably, the inverse correlation between pain and self-efficacy suggests that individuals experiencing greater pain tend to have lower confidence in their ability to manage symptoms or maintain activity levels. This psychological vulnerability increases the risk of poor compliance and functional decline^86,89^. Additionally, the association between pain and depressive symptoms, albeit modest, underscores the two-way relationship between persistent pain and emotional health.

### Function and psychological outcomes after TKA in individuals with knee OA

Functional recovery, measured using the LEFS, also demonstrated strong associations with psychological variables. Lower LEFS scores were correlated with greater kinesiophobia, pain catastrophizing, and central sensitization, suggesting that individuals reporting greater psychological distress experienced more difficulty in resuming physical function. These results are consistent with prior studies showing that maladaptive beliefs and emotional distress can reduce movement confidence, limit gait efficiency, and promote disuse^63,75,78^.

Furthermore, LEFS scores were inversely correlated with depression and perceived stress, reflecting how psychological strain may affect motivation, engagement in therapy, and neuromuscular recovery. On the other hand, higher self-efficacy was positively associated with functional scores, reinforcing its role as a protective factor against physical independence and sustained participation in rehabilitation activities^53,77,86^. These findings collectively point to the importance of psychological resilience in functional rehabilitation after TKA.

### Quality of life and psychological outcomes after TKA in individuals with knee OA

The relationship between quality of life and psychological outcomes was robust across both disease-specific (KOOS) and general health-related (SF-36) instruments. The KOOS subdomains, particularly those pertaining to pain, activities of daily living, and knee-related quality of life, were negatively associated with fear of movement, catastrophizing, and central sensitization. This suggests that psychological distress, even in the absence of overt physical complications, can substantially degrade perceived quality of life. These results align with evidence indicating that cognitive and emotional states influence how patients interpret disability^44,78^.

The SF-36 further demonstrated the broader impact of psychological health on overall well-being. Both the physical and mental component summary scores were statistically significantly influenced by depression, stress, and low self-efficacy, with the mental component showing the strongest inverse relationship with emotional distress. These results support the notion that long-term quality of life outcomes are not solely determined by surgical success or biomechanical correction but are also influenced by an individual’s emotional state and coping strategies^15,85^.

Importantly, arthritis self-efficacy emerged as a consistent and positive correlate of better quality of life across both the KOOS and the SF-36. This highlights the central role of self-belief in managing arthritis symptoms and sustaining long-term recovery. As a modifiable psychological resource, self-efficacy can be enhanced through structured education, goal setting, and psychological coaching, with the potential to significantly improve postoperative outcomes^25,86^.

### Socioeconomic, environmental, and occupational factors after TKA in individuals with knee OA

Socioeconomic and environmental factors were examined for their influence on long-term outcomes after TKA. Health insurance status, urban versus rural residence, and education level showed no significant associations with pain, function, quality of life, or psychological outcomes. Distance from the hospital showed a weak negative correlation with SF-36 scores, suggesting that greater travel distance may slightly affect overall quality of life^51^.

Occupational background influenced functional outcomes according to physical demands. Individuals engaged in physically demanding occupations, such as agriculture, manual labor, or shopkeeping, tended to have slightly lower functional scores compared to those in less physically demanding roles. This suggests that occupational demands may modestly influence long-term functional recovery, likely due to sustained mechanical loading or the need to resume physically intensive tasks, which could exacerbate residual limitations in mobility or strength. These observations underscore the importance of tailoring rehabilitation programs to account for patients’ occupational requirements, ensuring that recovery strategies address both functional independence and the demands of work.

These findings indicate that psychological factors such as kinesiophobia, pain catastrophizing, central sensitization, depression, perceived stress, and low self-efficacy are the primary determinants of recovery, while traditional socioeconomic indicators have minimal impact. The weak association with travel distance emphasizes the potential benefit of flexible follow-up strategies, including home-based care or tele-rehabilitation, for patients living farther from the hospital. Collectively, long-term recovery after TKA appears to be driven predominantly by biopsychosocial factors, with occupational demands contributing a modest but relevant influence on functional outcomes.

### Clinical implications

Even if pain is reduced post-TKA, mobility is not fully achieved in many patients. Most individuals tend to compensate by using other muscles and trick movements during functional activities, which can lead to altered biomechanics and secondary issues. Therefore, despite pain reduction, TKA may still significantly affect functional activities and overall quality of life. Psychological factors such as kinesiophobia, pain catastrophizing, central sensitization, depression, perceived stress and low self-efficacy may further exacerbate these limitations by reducing participation in rehabilitation and encouraging maladaptive movement patterns. These psychological barriers can limit the effectiveness of physical recovery and impede the achievement of optimal functional outcomes.

Regular follow-up plays a crucial role not only in monitoring implant performance and identifying emerging physical issues but also in evaluating the patient’s psychological status. Early detection of depressive symptoms, stress, or fear of movement allows healthcare providers to implement timely psychological and rehabilitative interventions. Addressing these factors holistically can enhance compliance, improve confidence in movement, and promote a more active lifestyle post-surgery, thereby increasing patient satisfaction and functional independence. Monitoring functional and social reintegration can help identify patients facing difficulty in daily life or community participation, allowing for early, individualized interventions.

Moreover, long-term follow-up provides valuable data that can guide clinical decision-making and research. Tracking both physical and psychological outcomes over time allows for the development of more targeted rehabilitation protocols and care pathways that address the full spectrum of recovery needs. Such approaches can reduce the risk of persistent pain, emotional distress, and functional decline. An integrated understanding of quality of life, functional status, and psychological well-being is essential for planning and developing comprehensive, long-term treatment strategies. Such strategies aim to minimize impairments, support early recovery, and facilitate long-term independence. Ultimately, considering psychological status as a core component of postoperative care helps improve long-term outcomes and ensures a patient-centric approach to TKA recovery.

The statistically significant associations of psychological factors with clinical outcomes identified in this study underscore the need to integrate psychological care into the TKA rehabilitation process. Addressing psychological factors such as fear of movement, pain catastrophizing, depressive symptoms, and low self-efficacy is essential for tailoring interventions to meet individual patient needs. The incorporation of psychological screening into routine clinical assessments can help identify patients at risk of poor recovery and enable early, targeted support. Interventions such as cognitive behavioral therapy, pain neuroscience education, stress management, and self-management training can effectively disrupt the cycle of chronic pain and physical inactivity and reduce quality of life. Adopting a comprehensive biopsychosocial approach throughout both preoperative counseling and postoperative rehabilitation has the potential to optimize recovery and promote long-term improvements in pain, physical function, emotional well-being, and overall life satisfaction following TKA.

### Limitations

Due to the retrospective design of the survey, preoperative data could not be included in the analysis. Although duration of preoperative symptoms was recorded, baseline psychological and pain severity measures were not available, limiting our ability to directly examine the influence of pain chronicity on long-term outcomes. Additionally, the reliance on self-report questionnaires may introduce response bias, as patients may underreport or overreport symptoms based on personal perceptions, memory, or social desirability. This may affect the accuracy of data regarding psychological status and functional outcomes. Furthermore, the cross-sectional design limits our ability to draw causal inferences between psychological factors and functional impairments post-TKA.

Longitudinal studies would be more informative in establishing the direction and strength of these associations over time. Nonrespondents were not significantly different in age or sex distribution from respondents, although selection bias cannot be ruled out. Another limitation is the inclusion of patients who underwent TKA across a broad time span, which may have introduced variability in surgical techniques, rehabilitation protocols, and clinical practices over the years. These differences could have influenced cumulative outcomes and potentially confounded the interpretation of the psychological and functional measures.

### Future recommendations

Future studies can focus on prospective long-term follow-up studies following TKA. Future research should explore the integration of psychological screening tools into standard postoperative care to better detect and manage issues such as the presence of kinesiophobia, pain catastrophizing, central sensitization, depression, stress and decreased self-efficacy, which can significantly impact long-term functional outcomes. Early detection of psychological barriers allows for timely implementation of supportive interventions. Moreover, the development and evaluation of multidisciplinary rehabilitation models are essential. These models should include coordinated care among physical therapists, psychologists, occupational therapists, and social workers. This team-based approach can address not only physical rehabilitation needs but also the emotional and social challenges faced by patients during recovery.

## Conclusion

This study evaluated long-term pain, functional status, quality of life, and psychological outcomes following total knee arthroplasty (TKA) in individuals with knee osteoarthritis. Although most participants reported overall improvement and satisfaction after surgery, a notable subset continued to experience persistent pain, functional limitations, and psychological distress. Significant associations between psychological factors and clinical outcomes reinforce the importance of a biopsychosocial approach in long-term postoperative care. Integrating psychological screening and individualized rehabilitation strategies into routine follow-up may help optimize recovery and enhance sustained physical and psychosocial well-being after TKA.

## Data Availability

The datasets used and/or analysed during the current study are available from the corresponding author on reasonable request.

## Abbreviations

TKA: Total knee arthroplasty
NPRS: Numerical pain rating scale
LEFS: Lower extremity functional scale
KOOS: Knee injury and osteoarthritis outcome score
SF-36: Short form-36 health survey questionnaire
PCS: Physical component summary
MCS: Mental component summary
IEC: Institutional ethics committee
CTRI: Clinical trial registry-India
MCID: Minimum clinically important difference
TSK: Tampa scale of kinesiophobia
PCS: Pain catastrophizing scale
CSI: Central sensitization inventory
PHQ-9: Patient health questionnaire-9
PSS: Perceived stress scale
ASES: Arthritis self-efficacy scale
